# Reference equations for evaluation of spirometry function tests in South Asia, and among South Asians living in other countries

**DOI:** 10.1183/13993003.02962-2021

**Published:** 2022-12-01

**Authors:** Wei Yee Leong, Ananya Gupta, Mehedi Hasan, Sara Mahmood, Samreen Siddiqui, Sajjad Ahmed, Ian Y. Goon, Marie Loh, Theresia H. Mina, Benjamin Lam, Yik Weng Yew, Joanne Ngeow, Jimmy Lee, Eng Sing Lee, Elio Riboli, Paul Elliott, Geak Poh Tan, Sanjay H. Chotirmall, Ananda R. Wickremasinghe, Jaspal S. Kooner, Khadija I. Khawaja, Prasad Katulanda, Malay K. Mridha, Sujeet Jha, Anjana Ranjit Mohan, Guha Pradeepa, Anuradhani Kasturiratne, John C. Chambers

**Affiliations:** 1 Lee Kong Chian School of Medicine, Nanyang Technological University, Singapore; 2 Department of Epidemiology and Biostatistics, School of Public Health, Imperial College London, London, UK; 3 Institute of Endocrinology, Diabetes and Metabolism, Max Super Speciality Hospital (Devki Devi Foundation), New Delhi, India; 4 Faculty of Medicine, Imperial College London, London, UK; 5 Centre for Non-communicable Disease and Nutrition (CNCDN), BRAC James P Grant of Public Health, BRAC University, Dhaka, Bangladesh; 6 Department of Endocrinology and Metabolism, Services Institute of Medical Sciences, Services Hospital, Lahore, Pakistan; 7 Punjab Institute of Cardiology, Punjab, Pakistan; 8 Tyree Foundation Institute of Health Engineering, The University of New South Wales, Sydney, Australia; 9 National Skin Centre, Singapore; 10 Department of Family and Community Medicine, Khoo Teck Puat Hospital, Singapore, Singapore; 11 Cancer Genetics Service, Division of Medical Oncology, National Cancer Centre Singapore, Singapore; 12 Institute of Mental Health, Singapore; 13 Clinical Research Unit, National Healthcare Group Polyclinics, Singapore; 14 MRC Centre for Environment and Health, Imperial College London, London, UK; 15 Department of Respiratory and Critical Care Medicine, Tan Tock Seng Hospital, Singapore; 16 Department of Public Health, Faculty of Medicine, University of Kelaniya, Ragama, Sri Lanka; 17 National Heart and Lung Institute, Imperial College London, London, UK; 18 Department of Clinical Medicine, Faculty of Medicine, University of Colombo, Colombo, Sri Lanka; 19 Madras Diabetes Research Foundation, Chennai, India

## Abstract

**Background:**

There are few data to support accurate interpretation of spirometry data in South Asia, a major global region with a high reported burden of chronic respiratory disease.

**Method:**

We measured lung function in 7453 healthy men and women aged ≥18 years, from Bangladesh, North India, South India, Pakistan and Sri Lanka, as part of the South Asia Biobank study. First, we assessed the accuracy of existing equations for predicting normal forced vital capacity (FVC), forced expiratory volume in 1 s (FEV_1_) and FEV_1_/FVC ratio. Then, we used our data to derive (n=5589) and internally validate (n=1864) new prediction equations among South Asians, with further external validation among 339 healthy South Asians living in Singapore.

**Results:**

The Global Lung Initiative (GLI) and National Health and Nutrition Examination Survey consistently overestimated expiratory volumes (best fit GLI-African American, mean±sd z-score: FEV_1_ −0.94±1.05, FVC −0.91±1.10; n=7453). Age, height and weight were strong predictors of lung function in our participants (p<0.001), and sex-specific reference equations using these three variables were highly accurate in both internal validation (z-scores: FEV_1_ 0.03±0.99, FVC 0.04±0.97, FEV_1_/FVC −0.03±0.99) and external validation (z-scores: FEV_1_ 0.31±0.99, FVC 0.24±0.97, FEV_1_/FVC 0.16±0.91). Further adjustment for study regions improves the model fit, with highest accuracy for estimation of region-specific lung function in South Asia.

**Conclusion:**

We present improved equations for predicting lung function in South Asians. These offer the opportunity to enhance diagnosis and management of acute and chronic lung diseases in this major global population.

## Abstract 2

**Newly derived spirometry reference equations greatly improve prediction of normal lung function in South Asians, thus filling the long-standing gap of appropriate reference values for this ethnic group** https://bit.ly/3H7ZGt5

## Introduction

Spirometry provides a simple noninvasive method for assessing pulmonary function, and is a key tool in the diagnosis of obstructive and restrictive lung diseases. Accurate interpretation of the results from spirometry testing requires knowledge of expected normal values in a healthy population. Since pulmonary function is influenced by physiological factors such as age, height, sex and weight, estimation of normal values is facilitated by reference equations that take into account an individual's relevant characteristics. Normative ranges for lung function also show striking differences between populations; with higher lung function among people of European ancestry, compared to African-American and Asian populations [1–4]. In a large cohort study (PURE), lung function was reported to be the lowest in South Asians compared to North Americans or Europeans, even after adjustment for height, age and sex [5]. These variations are not limited to differences between regions, but are evident within countries where there are distinct ethnic subgroups [6]. Thus, the ethnic background of an individual is an important consideration for interpretation of lung function results. [7, 8] Use of inaccurate normative ranges may lead to misclassification, over- and under-diagnosis and inappropriate treatment, all of which can adversely affect healthcare outcomes.

The Global Lung Initiative (GLI 2012) [9] and the National Health and Nutrition Examination Survey (NHANES III) [10] have been the primary datasets used for development of reference equations to describe normative ranges. While these datasets have large sample sizes for people of European and African heritage, other ethnic groups are less well represented. In particular, there are few data to inform normative ranges for South Asia, a region that is home to 25% of the world's population, and with a high reported burden of chronic respiratory disease [11]. As a consequence, the GLI 2012 and other leading reference equations for sprirometry do not take South Asian ethnicity into account, and the validity of the GLI 2012 and other international equations is not known in this major global population. The purpose of this study was to address this health need, and to derive predictive equations that are more accurate in estimating normal lung function in South Asian individuals.

## Methods

Our study design is summarised in supplementary figure S1. In brief, we first evaluated the performance of the GLI 2012 and NHANES III spirometry equations using the data from 43 472 participants in the South Asia Biobank study. Next, we identified and evaluated reference equations reported for South Asians in available, published studies. Finally, we used our data to develop a South Asian specific spirometry equation, with both internal and external validation, including among people of South Asian ancestry who were born or living overseas.

### Study populations

#### The South Asia Biobank study

This is an ongoing prospective study comprising a representative, population-based cohort of >100 000 people aged ≥18 years, recruited from 118 surveillance sites that that were centred on local primary community healthcare units in five study regions: Bangladesh, South India, North India, Pakistan and Sri Lanka. Spirometry data were collected as a part of respiratory evaluation for environmental and smoking-related lung injury. Recruitment started in November 2018; the study method has been described previously [12]. We used data from subjects recruited prior to February 2020 in the current analysis (n=43 472; selected prior to the coronavirus disease 2019 (COVID-19) pandemic). The distribution of subjects by study region is shown in table 1. Subjects were excluded if they were current or ex-smokers, had a self-reported history of cardiovascular and lung disease and presence of respiratory symptoms (shortness of breath, persistent cough for ≥2 weeks, sputum during coughing, blood in sputum, wheezing or early morning cough with chest tightness) in the past 6 months, or if their spirometry results did not meet the acceptability criteria according to the American Thoracic Society (ATS)/European Respiratory Society (ERS) standards [13]. Supplementary table S1 presents the number of subjects by exclusion criteria. 7453 subjects were included in the final analysis. The South Asia Biobank study was approved by the Imperial College London research ethics committee (18IC4698), and all participants provided informed consent.

**TABLE 1:** Baseline characteristics of study participants by sex and study region

	**Bangladesh**	**North India**	**South India**	**Pakistan**	**Sri Lanka**	**Total**
**Females**						
Participants	1968	1471	358	217	1463	5477
Age^#^ (years)	42.2±12.5	41.7±12.1	50.6±12.3	44.1±11.1	49.1±12.4	44.5±12.8
Height^¶^ (cm)	150.3±5.6	151.7±5.5	150.0±6.4	154.4±6.9	152.8±5.9	151.5±5.9
Weight^+^ (kg)	54.2±11.1	60.6±12.9	63.0±14.8	73.5±15.9	62.5±11.6	59.5±13.1
FEV_1_ (L)	1.90±0.40	1.79±0.41	1.46±0.44	1.78±0.44	1.79±0.42	1.81±0.43
FVC (L)	2.32±0.47	2.21±0.48	1.71±0.57	2.21±0.54	2.14±0.51	2.20±0.51
FEV_1_/FVC (%)	81.8±4.9	80.8±5.2	86.9±8.6	80.7±7.4	83.9±5.9	82.4±5.9
**Males**						
Participants	640	672	161	75	428	1976
Age^#^ (years)	43.1±12.3	42.3±13.0	50.7±12.1	44.5±11.5	49.9±13.2	45.0±13.1
Height^¶^ (cm)	162.3±6.5	164.3±6.8	162.9±8.2	167.9±7.3	165.4±6.8	163.9±7.0
Weight^+^ (kg)	61.2±12.2	68.5±13.6	69.5±16.4	79.5±17.7	69.7±13.5	66.9±14.3
FEV_1_ (L)	2.65±0.54	2.53±0.61	2.12±0.63	2.62±0.58	2.56±0.56	2.55±0.59
FVC (L)	3.30±0.63	3.16±0.71	2.59±0.75	3.32±0.70	3.13±0.64	3.16±0.70
FEV_1_/FVC (%)	80.5±5.8	79.8±6.1	82.0±8.1	79.0±8.0	81.7±6.7	80.6±6.4

Data are presented as n or mean±sd. n=7453. FEV_1_: forced expiratory volume in 1 s; FVC: forced vital capacity. ^#^: range 18–85 years (female and male); ^¶^: range 122–175.0 cm (female), 128.0–187.0 cm (male); ^+^: range 23–157.9 kg (female), 33.4–180.5 kg (male).

Data were collected using a single, standardised protocol at all study sites. An interviewer-administered questionnaire was used to collect sociodemographic data, information on smoking habit and respiratory and cardiovascular health. Height was measured using a portable stadiometer (SECA213), and weight using electronic scales (OMRON BF-511). Lung function testing was performed according to ATS/ERS guidelines [13], using the Air Next spirometer (NUvoAir, Sweden) device, a portable hand-held spirometer with disposable precalibrated turbine tacograph connected wirelessly to the mobile application AirMD, where data are stored initially. This device has been validated previously [14–16] and meets the ATS/ERS requirements [17]. The study technician first explained the spirometry manoeuvre to the subject, and provided a demonstration using an instructional video produced by the manufacturer. Participants were asked to perform a minimum of three, up to a maximum of six manoeuvres, to obtain three successful measurements. The NuvoAir app uses a grading system recommended by the ATS/ERS for spirometer reporting. Acceptability and reproducibility of the spirometry tests were graded in accordance with the ATS/ERS guidelines [13]. Only spirometry measurements with forced expiratory volume in 1s (FEV_1_) and forced vital capacity (FVC) of at least grade C were included. Only measurements that met these criteria were included, of which the largest FVC and FEV_1_ values were selected for analysis. All data were collected using a shared cloud-based database; spirometry data were captured at source by device integration.

#### The HELIOS study

This is a longitudinal study of a multi-ethnic population in Singapore (www.healthforlife.sg/). HELIOS (Health for Life in Singapore) inclusion criteria specify Singaporean citizens or permanent residents aged 30–84 years. For the present external validity study, we identified 710 people of self-reported South Asian ancestry from among the 6400 people recruited into the HELIOS study prior to March 2020 (onset of the COVID-19 pandemic). From these, we selected 339 healthy never-smokers (63.1% female) with an age range of 30–81 years (supplementary figure S1). Sociodemographic, lifestyle and health information, including smoking habit, respiratory and cardiovascular health were collected by questionnaire. Lung function test was performed according to ATS/ERS guidelines [11], using the MIR Spirolab (Rome, Italy), with the accompanying Winspiro PRO software.

### Data analysis

All categorical data are presented as frequencies or proportion (%) and continuous variables are presented as mean±sd or mean (95% CI). All statistical analyses were conducted using STATA (version 14; StataCorp, College Station, TX, USA).

Considering their wide usage in clinical laboratories and spirometer manufacturers, we first evaluated the performance of GLI 2012 [9] and NHANES III [10] reference equations on our healthy, never-smoker study participants (n=7453), by comparing their mean z-scores and percentage of subjects with z-scores <−1.645, also defined as lower limit of normal (LLN). Deviation of ≥0.5 from the mean z-score of 0 was considered clinically significant, in line with previous studies [18–21]. Analysis of z-scores was carried out in men and women separately, and stratified by age. For NHANES III, we evaluated “Caucasian” and “African-American”; for GLI 2012 we evaluated four of the available ethnic reference categories: “others/mixed”, “African-American”, “Caucasian” and “South-East Asian” in our study population. The best-fitting ethnic group will then be applied for the subsequent comparison analysis. We also compared the evaluation with eight published South Asian reference equations (since 2005, when the ATS/ERS guidelines were published [17]): Saleem *et al*. [22] (SAL), Chhabra *et al*. [23], Dasgupta *et al*. [24], Desai *et al*. [25], Biswas *et al*. [26], Agarwal *et al*. [27], Sooriyakanthan *et al*. [28] and Memon *et al*. [29]. Supplementary tables S2 and S3 summarise the list of publications and their reference equations that were considered for inclusion in this study.

We then used the data to derive new reference equations that might more accurately predict pulmonary function in healthy South Asians. We divided the South Asian Biobank study data at random (3:1) into development (n=5589) and internal validation (n=1864) datasets. With the development dataset, we use multiple linear regression to develop new predictive equations for FEV_1_, FVC and FEV_1_/FVC ratio in each sex. We considered three modelling strategies that include age, height, weight and study regions as covariates. The first model (M1) included only age and height as the independent variables, to provide consistency with GLI 2012 and NHANES III equations. There was no evidence of nonlinear relationships and using μ-σ-λ analysis approaches did not materially improve the degree of fit for any of our analyses. For our second model (M2), we included weight in addition to age and height, as weight has been shown to be a potential predictor for lung function [30]. We then additionally considered study regions (as a factor variable) in our third model, (M3) to adjust for the variability in spirometry measurements between different countries in South Asia. When developing regression models, we used polynomial functions to exclude nonlinear relationships between age and lung function measures. Predictive accuracy of these derived equations were subsequently assessed in the validation dataset (n=1864) and an independent external dataset of South Asians born or living overseas (HELIOS study, n=339). The GLI 2012, NHANES III and other South Asian equations were also included for comparison in analyses of both the internal and external validation datasets.

To assess their predictive performance, we calculated predicted FEV_1,_ FVC and FEV_1_/FVC ratio for all participants, as well as the corresponding expected LLN, using each of the reference equations identified. We then determined the difference between observed and predicted, expressed as percentage predicted, z-score and the proportion of observed values falling below the predicted LLN using the following formula:

% predicted=observed/predicted×100

LLN=predicted – (1.645×residual standard deviation)

z-score=(observed – predicted)/residual standard deviation

To evaluate the possible misclassification of pulmonary function test results, we also classified individuals as showing obstructive, restrictive or mixed ventilatory defect based on accepted criteria (obstructive: FEV_1_/FVC<LLN and FVC≥LLN; restrictive: FEV_1_/FVC≥LLN and FVC<LLN; mixed: FEV_1_/FVC<LLN and FVC<LLN; and normal: FEV_1_/FVC≥LLN and FVC≥LLN) [31].

## Results

### Study participants

The characteristics of the 7453 healthy participants selected from among the South Asia Biobank study are summarised in table 1. 73.5% of our study population were female with a mean±sd (range) age of 44.5±12.8 (18–85) years. Between countries, people from Pakistan were taller and heavier, while Sri Lankan subjects were older compared to those from other settings. FEV_1_ and FVC were highest in Bangladesh and lowest in India, while FEV_1_/FVC ratio was highest in Sri Lanka (detailed comparisons, with statistical significance, are shown in supplementary table S4). Baseline characteristics of external HELIOS participants are summarised in supplementary table S5. They are on average older, taller and heavier than the South Asia Biobank participants, with higher FEV_1_ and FVC measurements.

### Evaluation of GLI 2012, NHANES III and other South Asian reference equations

**FIGURE 1:**
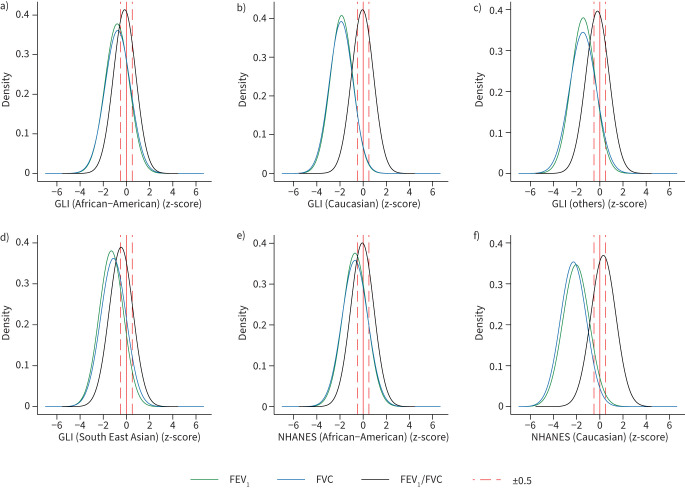
Density plots of z-scores for forced expiratory volume in 1 s (FEV_1_), forced vital capacity (FVC) and FEV_1_/FVC in males using a–d) Global Lung Initiative (GLI) 2012 and e, f) National Health and Nutrition Examination Survey (NHANES) III reference values for different ethnic groups (n=7453).

**FIGURE 2:**
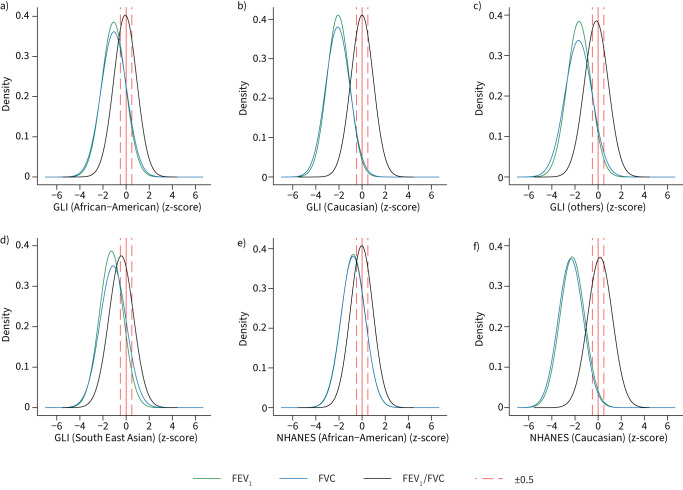
Density plots of z-scores for forced expiratory volume in 1 s (FEV_1_), forced vital capacity (FVC) and FEV_1_/FVC in females using a–d) Global Lung Initiative (GLI) 2012 and e, f) National Health and Nutrition Examination Survey (NHANES) III reference values for different ethnic groups (n=7453).

**FIGURE 3:**
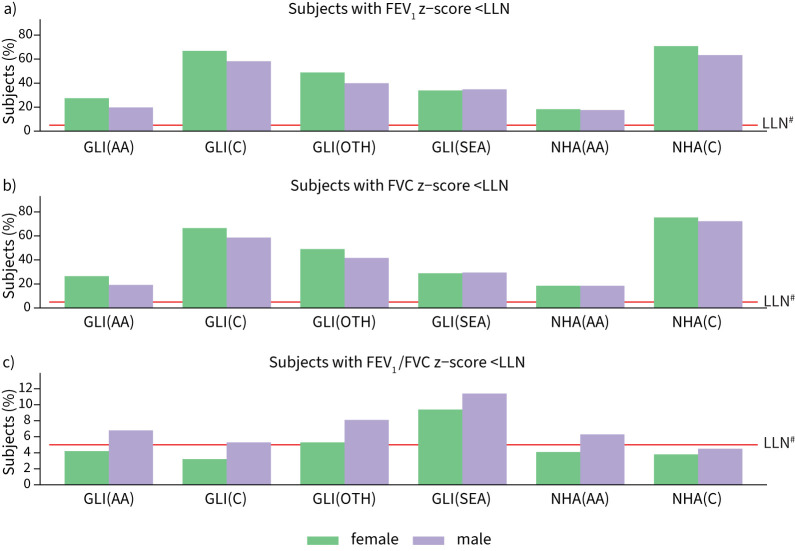
Percentage of healthy subjects categorised as below lower limit of normal (LLN) in a) forced expiratory volume in 1 s (FEV_1_), b) forced vital capacity (FVC) and c) FEV_1_/FVC using Global Lung Initiative (GLI) 2012 and National Health and Nutrition Examination Survey (NHANES) III reference values for different ethnic groups, stratified by gender (n=7453). GLI(AA): GLI 2012 (African-American); GLI(C): GLI 2012 (Caucasian); GLI(OTH): GLI 2012 (others); GLI(SEA): GLI 2012 (South-East Asian); NHA(AA): NHANES III (African-American); NHA(C): NHANES III (Caucasian). ^#^: the expected LLN in a healthy population at 5%.

Figures 1 and 2 show the density plot of z-scores for FEV_1_ and FVC values in South Asians predicted by the GLI 2012 and NHANES III equations in men and women respectively. There is a shift for all reference values to lower z-scores (>−0.5) in men and women (p<0.0001; supplementary table S6), which are considered clinically significant and correspond to ≥5–6% difference in the lung measurement [9, 20, 32]. The differences were greatest using the Caucasian reference population and lowest in African-Americans. Both the GLI 2012 and NHANES III equations also identified a high proportion of our healthy South Asian participants as having pulmonary function test values below LLN. ≥50% were identified to be below the LLN of FEV_1_ and FVC using Caucasian reference equations, and >17% were still below LLN using the African-American reference values, in both men and women (figure 3). For FEV_1_/FVC, the mean z-scores are closer to 0, with <11% of subjects below the LLN across all ethnic groups and sex.

We also found wide variation between observed and predicted values using published reference equations from South Asian countries. Although mean z-scores for FEV_1_ and FVC were higher than GLI 2012 and NHANES III (except for SAL) in both men and women, the differences between observed and expected were all statistically significant (p<0.01). 0.7–59.8% of our healthy subjects (both men and women) were categorised as below the LLN of FEV_1_ and FVC (supplementary table S7).

### Development and evaluation of reference equations specific for South Asia

The characteristics of participants used for development and internal validation for prediction of normal lung function in South Asians are shown in supplementary table S8. There are no significant differences between the development and internal validation cohort. Table 2 summarises the predictive models developed by regression analysis among men and women in our discovery sample. Model fit was improved with incorporation of additional predictors, with highest R^2^ for model M3 (supplementary table S9).

**TABLE 2:** Derived predictive equations for forced expiratory volume in 1 s (FEV_1_), forced vital capacity (FVC) and FEV_1_/FVC stratified by sex using the training dataset (n=5589; female n=4121, male n=1468).

	**Predictive equations**	**R^2^**	**RSD**
**M1**			
Female			
FEV_1_ (L)	−1.027−0.0173(age)+0.0238(height)	0.429	0.323
FVC (L)	−1.533−0.0200(age)+0.0305(height)	0.422	0.392
FEV_1_/FVC	0.929−0.000209(age)−0.000628(height)	0.005	0.004
Male			
FEV_1_ (L)	−2.225−0.0215(age)+0.0350(height)	0.429	0.449
FVC (L)	−3.349−0.0224(age)+0.0458(height)	0.422	0.531
FEV_1_/FVC	0.976−0.001221(age)−0.000703(height)	0.063	0.062
**M2**			
Female			
FEV_1_ (L)	−1.152−0.0172(age)+0.0251(height)−0.00139(weight)	0.430	0.322
FVC (L)	−1.883–0.0199(age)+0.0343(height)–0.00390(weight)	0.430	0.430
FEV_1_/FVC	1.008–0.000229(age)–0.00149(height)+0.000877(weight)	0.034	0.003
Male			
FEV_1_ (L)	−2.507–0.0215(age)+0.0379(height)–0.00295(weight)	0.432	0.448
FVC (L)	−3.902−0.0224(age)+0.0516(height)−0.00575(weight)	0.433	0.526
FEV_1_/FVC	1.035–0.00122(age)–0.00132(height)+0.000614(weight)	0.077	0.062
**M3**			
Female			
FEV_1_ (L)	−1.060 −0.0167(age)+0.0242(height)+0.0005(weight)–0.169(North India) −0.296(South India)−0.204(Pakistan)−0.0679(Sri Lanka)	0.471	0.311
FVC (L)	−1.859−0.0185(age)+0.0336(height)−0.00148(weight)−0.172(North India)−0.432(South India)−0.199(Pakistan)−0.139(Sri Lanka)	0.473	0.375
FEV_1_/FVC	1.039−0.000605(age)−0.00158(height)+0.000765(weight)−0.0123(North India)+0.0473(South India)−0.0152(Pakistan)−0.0246(Sri Lanka)	0.118	0.056
Male			
FEV_1_ (L)	−2.303−0.021(age)+0.0365(height)−0.00109(weight)−0.199(North India)−0.404(South India)−0.162(Pakistan)−0.0604(Sri Lanka)	0.471	0.432
FVC (L)	−3.704−0.0211(age)+0.0500(height)−0.00347(weight)−0.212(North India)−0.558(South India)−0.129(Pakistan)−0.144(Sri Lanka)	0.476	0.506
FEV_1_/FVC	1.049−0.00145(age)−0.00134(height)+0.000599(weight)−0.00988(North India)+0.0214(South India)−0.0172(Pakistan)+0.0198(Sri Lanka)	0.114	0.061

RSD: residual standard deviation; M1: age and height as independent variables; M2: M1 + weight; M3: M2 + study regions.

**FIGURE 4:**
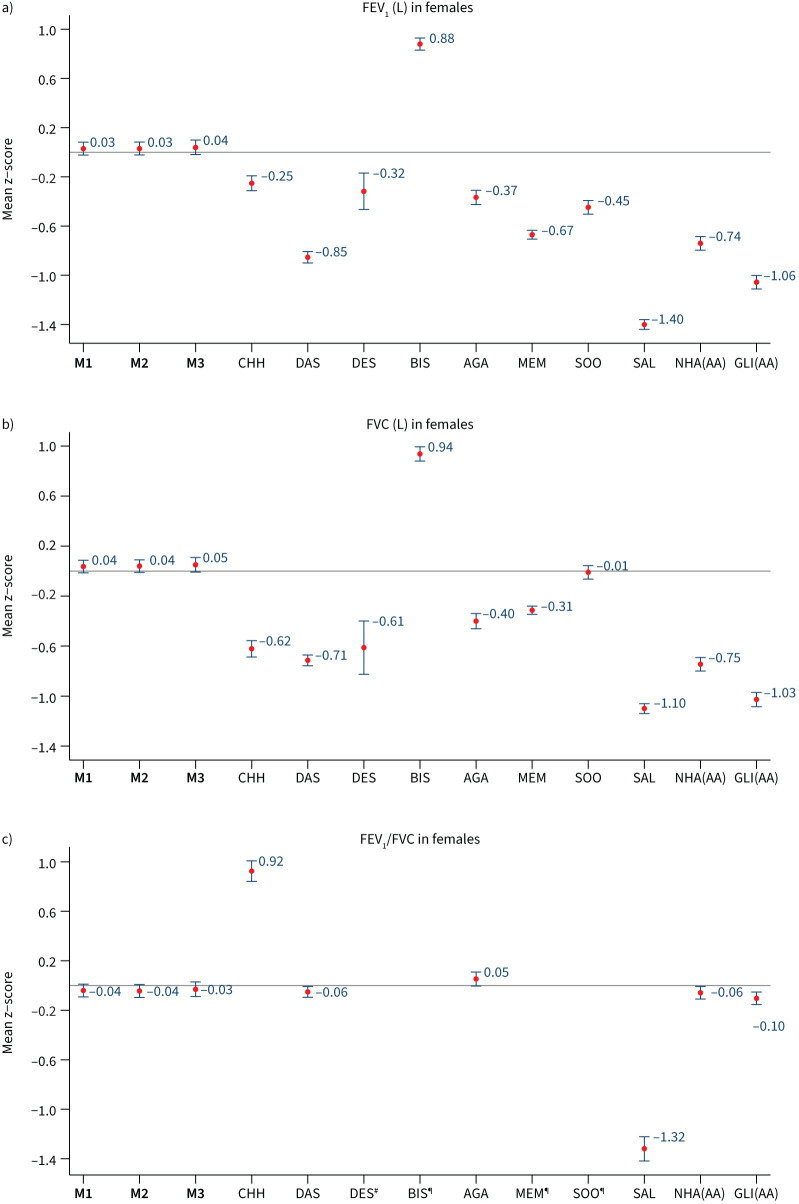
Internal validation (n=1356). Mean (95% confidence interval) of z-scores for a) forced expiratory volume in 1 s (FEV_1_), b) forced vital capacity (FVC) and c) FEV_1_/FVC in females, derived from different predictive equations. M1, M2, M3: predictive equations derived from current study; CHH: Chhabra [23]; DAS: Dasgupta [24]; DES: Desai [25]; BIS: Biswas [26]; AGA: Agarwal [27]; MEM: Memon [29]; SOO: Sooriyakanthan [28]; SAL: Saleem [22]; NHA(AA): National Health and Nutrition Examination Survey III (African-American); GLI(AA): Global Lung Initiative 2012 (African-American) ^#^: results not shown because the value is off-scale (mean z-score −5.09, 95% CI −5.17–−5.02); ^¶^: results for BIS, MEM and SOO are not available.

**FIGURE 5:**
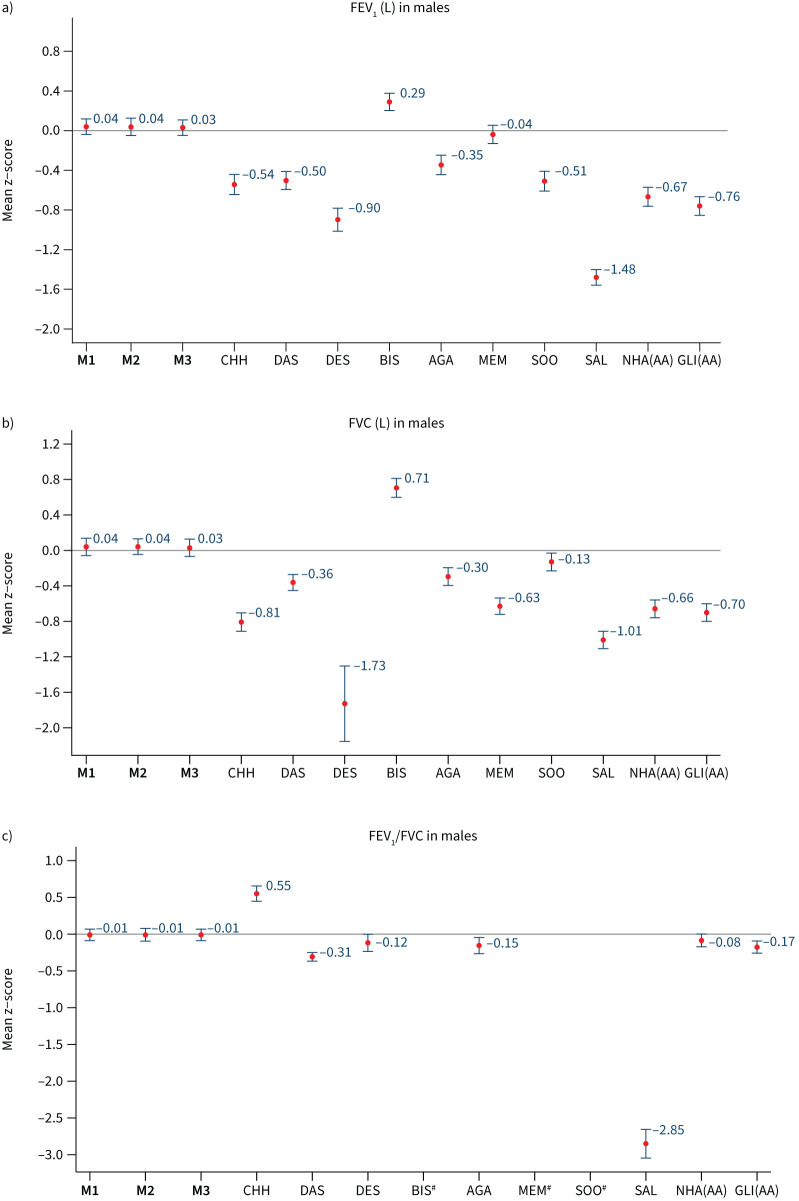
Internal validation (n=508). Mean (95% confidence interval) z-scores for a) forced expiratory volume in 1 s (FEV_1_), b) forced vital capacity (FVC) and c) FEV_1_/FVC in males, derived from different predictive equations. M1, M2, M3: predictive equations derived from current study; CHH: Chhabra [23]; DAS: Dasgupta [24]; DES: Desai [25]; BIS: Biswas [26]; AGA: Agarwal [27]; MEM: Memon [29]; SOO: Sooriyakanthan [28]; SAL: Saleem [22]; NHA(AA): National Health and Nutrition Examination Survey III (African-American); GLI(AA): Global Lung Initiative 2012 (African-American) ^#^: results for BIS, MEM and SOO are not available.

When we evaluated our prediction models among participants in our internal validation dataset (n=1864), models M1, M2 and M3 all showed high concordance between observed and predicted FEV_1_, FVC and FEV_1_/FVC. Among all participants, mean±sd z-scores for FEV_1_ were 0.03±0.98, 0.03±0.09 and 0.04±1.00 in females (figure 4), and 0.04±1.01, 0.04±1.02 and 0.03±1.01 in males (figure 5); using models M1, M2 and M3, respectively. Similar, but larger, differences were reported for FVC. Results were consistent when analyses were stratified by country (supplementary table S10) and age group (supplementary figure S2), with mean z-scores between −0.5 and 0.5 and observed values that were within 5–6% predicted for all spirometry indices. Overall, M3 displayed better predictive accuracy for region-specific results (supplementary table S10).

Compared to our derived reference equations, GLI 2012, NHANES III and other published South Asian specific models had lower predictive accuracy. Their mean z-scores for FEV_1_ and FVC showed greater deviation from 0, indicating a poor fit to our study population. The results are similar when stratified by age group (supplementary figures S2 and S3). Among the internal validation samples, GLI 2012 and NHANES III continued to significantly overpredict FEV_1_ and FVC for both men and women, and the reported South Asian functions were consistently less accurate than our derived reference equation. The calculated LLN for these equations resulted in a high number of subjects being identified as below their LLN, which deviated from the expected 5% in a healthy population (supplementary table S11). This leads to a high fraction of healthy participants classified as having abnormal lung function with GLI 2012 and South Asian reference equations, compared to <10% with our derived reference values (supplementary table S12).

### External validation (HELIOS dataset)

**FIGURE 6:**
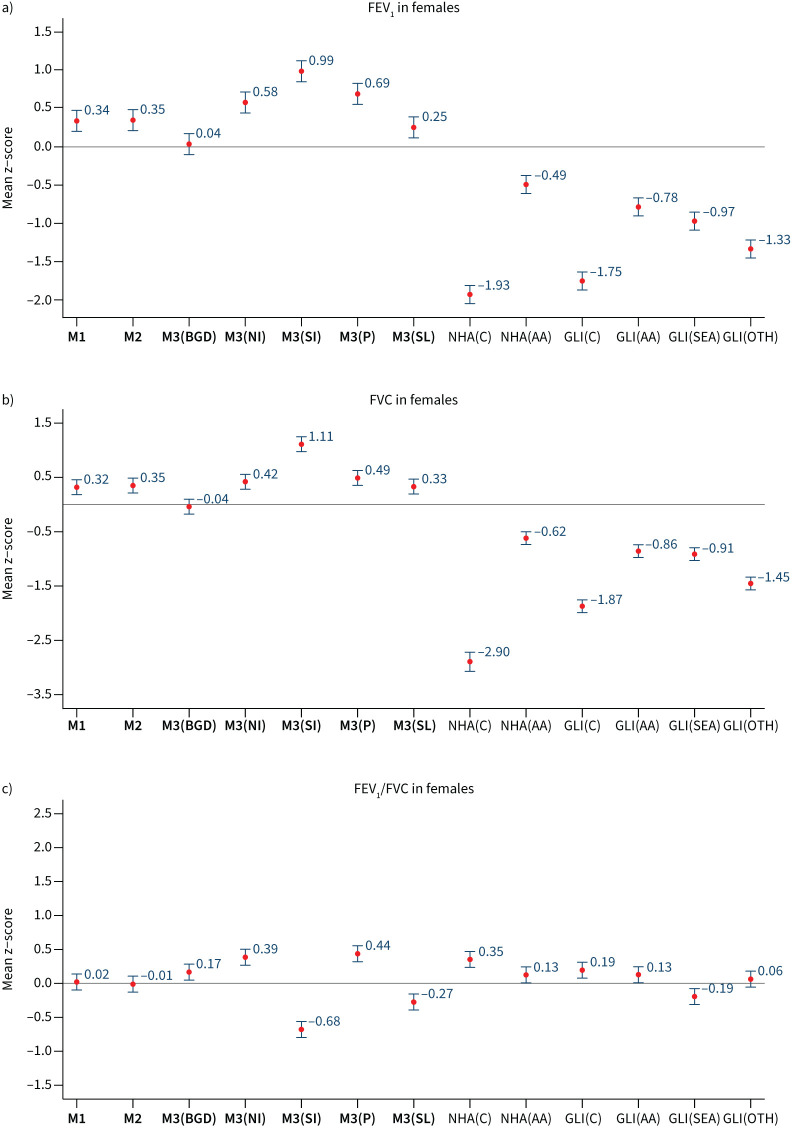
External validation (Health for Life in Singapore (HELIOS) data, n=214). Mean (95% confidence interval) z-scores for a) forced expiratory volume in 1 s (FEV_1_), b) forced vital capacity (FVC) and c) FEV_1_/FVC in females, derived from different predictive equations. M3(BGD): Bangladesh; M3(NI): North India; M3(SI): South India; M3(P): Pakistan; M3(SL): Sri Lanka; NHA(C): National Health and Nutrition Examination Survey (NHANES) III (Caucasian). NHANES and Global Lung Initiative (GLI) 2012 reference values: NHA(AA): NHANES(African-American); GLI(C): GLI(Caucasian); GLI(AA): GLI(African-American); GLI(SEA): GLI(South-East Asia); GLI(OTH): GLI(others).

**FIGURE 7:**
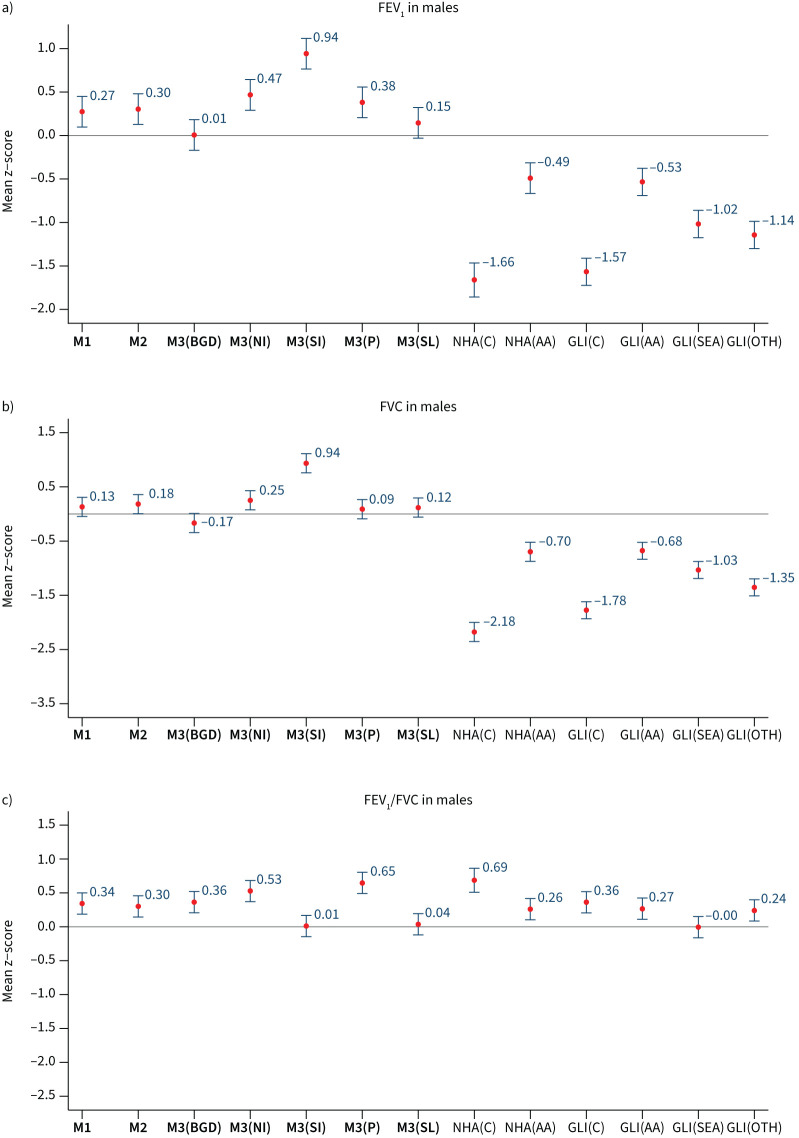
External validation (Health for Life in Singapore (HELIOS) data, n=125). Mean (95% confidence interval) z-scores for a) forced expiratory volume in 1 s (FEV_1_), b) forced vital capacity (FVC) and c) FEV_1_/FVC in males, derived from different predictive equations. M3(BGD): Bangladesh; M3(NI): North India; M3(SI): South India; M3(P): Pakistan; M3(SL): Sri Lanka; NHA(C): National Health and Nutrition Examination Survey (NHANES) III (Caucasian). NHANES and Global Lung Initiative (GLI) 2012 reference values: NHA(AA): NHANES(African-American); GLI(C): GLI(Caucasian); GLI(AA): GLI(African-American); GLI(SEA): GLI(South-East Asia); GLI(OTH): GLI(others).

We evaluated our M1 and M2 reference equations among our external validation set comprising 339 people of South Asian ancestry living in Singapore. Figures 6 and 7 show the mean z-scores for each reference equation, in women and men, respectively. Mean±sd z-scores for M1 and M2 equations ranged from 0.13±0.99 to 0.34±0.96 for FEV_1_ and FVC. Similar to observations in the South Asia Biobank cohort, model fit was poor for FEV_1_ and FVC with GLI 2012 and NHANES III. In addition, models M1 and M2 predicted FEV_1_/FVC accurately in the HELIOS cohort, with z-scores ranging from −0.01±0.90 to 0.34±0.91, as did the GLI (South-East Asia) equations (mean z-score <|0.2|). Among our region-specific models, the M3 equation for Bangladesh and Sri Lanka had good fit for predicting FEV_1,_ FVC and FEV_1_/FVC, compared to other study regions, with percentage below LLN close to 5% (supplementary table S13).

## Discussion

International reference equations such as NHANES III and GLI 2012 are widely adopted as the preferred standard for prediction of normal lung function indices, and are commonly included in spirometry devices to enable rapid or automated interpretation of results. However, at the time these equations were developed, large-scale data were not available for South Asians, and the validity of these functions in this major ethnic group is unclear. Although there are locally derived reference equations for different subsets of South Asian population, their development has been limited to single-centre studies, without validation to assess generalisability [33].

In our study, we find that both the GLI 2012 and NHANES III equations substantially overpredict FEV_1_ and FVC in men and women. The best-fitting ethnic reference group from GLI 2012 is African-Americans, but this still overestimates FEV_1_ and FVC, with ≥20% of our healthy subjects classified as having abnormal lung function. These findings are consistent with other studies conducted in South Asia [34–36]. A similar trend was observed using published reference equations developed in South Asian populations. The available reference equations are thus inaccurate in South Asians, and overestimate the proportion of people who have restrictive ventilatory defect by at least three-fold. This might lead to unnecessary and expensive further investigations, and potentially inappropriate treatment.

It is well established that lung function varies between global regions and ethnic groups, in part reflecting anthropometric differences such as chest dimension and adiposity and lung size [37]. Socioeconomic factors, habitual levels of physical activity, environmental exposures and genetic background are also likely to be important. Observations that lung function varies between ethnic groups emphasises the need for reference equations that are developed and validated for the target population.

To advance beyond the status quo, we have developed new sex-specific risk functions for prediction of lung function in South Asians. Our simplest model includes just age and height, in keeping with international practice. Weight and study region were predictors of lung function independently of age and height, and including these variables into the prediction models improved the variance explained. Thus, we also present models that include weight and study region to enable the most accurate, region-specific estimation of lung function. While all three models showed good predictive accuracy, the M3 models were found to be the most accurate and are thus recommended for clinical purposes in people living in their respective South Asian regions.

We used data from the HELIOS study for external validation of our equations in South Asian people. We show that our generalised models that do not include region (M1 and M2) are more accurate for both FEV_1_ and FVC than other reported normative equations, including all available GLI models. FEV_1_/FVC was also predicted accurately by our M1 and M2 equations, although the GLI (South-East Asia) equation showed best overall fit. In addition, we explored our region-specific M3 equations in the HELIOS study population and show that Sri Lanka reference equations gave a better fit for predicting FEV_1_, FVC and FEV_1_/FVC than did the other study regions. This is consistent with the majority of the Singapore Indian ethnic group being descendants of Tamil settlers from South Asia. Thus, our normative reference equations are highly accurate in both internal and external validation, including among a cohort of South Asians born or living overseas, and whose lung function was measured with a different spirometer. These optimised reference equations offer the prospect of more accurate diagnosis and improved healthcare for South Asians, who represent 25% of the global population.

The strengths of our study include large sample size with standardised protocol and spirometer devices being used across different countries, compared to the existing studies that were mostly derived from relatively small sample sizes with variations in instruments and exclusion criteria [31]. Our study population includes South Asians from multiple geographic regions, with people of both North and South Indian ancestry, thus capturing both ends of the primary genetic axis of the Indian subcontinent [38]. We adhered to the latest ATS/ERS guidelines in interpreting our spirometry results, such as defining LLN based on statistical calculation instead of fixed percentage. Furthermore, our derived reference values showed high internal and external validity, reflecting the robustness of the predicitive equations.

There are some limitations. Firstly, subjects from Pakistan were under-represented. Future studies with higher sampling of subjects from Pakistan will be helpful to improve the precision of our algorithms. Secondly, respiratory symptoms and comorbidities for exclusion criteria were based on survey questionnaires and might not have excluded all people with chronic diseases. Nevertheless, we adhered to common exclusion criteria that are applied in most spirometry studies and taking into consideration that individuals with certain comorbidities do exist in the general healthy population. Thirdly, countries such as Afghanistan, Bhutan, Nepal and Maldives were not included in the study, although they are part of the wider South Asia region. Further validation in these subpopulations will be useful to determine the generalisability and representation of our new equations in South Asia. Our study provides an updated spirometry reference values for the South Asian adult population. These newly derived reference equations will improve the accuracy of lung function predictions in the South Asian population, reducing misclassification and improving diagnosis and healthcare for this major global population.

## Supplementary material

10.1183/13993003.02962-2021.Supp1 **Please note:** supplementary material is not edited by the Editorial Office, and is uploaded as it has been supplied by the author.
Supplementary material ERJ-02962-2021.Supplement

## Shareable PDF

10.1183/13993003.02962-2021.Shareable1 This one-page PDF can be shared freely online.
Shareable PDF ERJ-02962-2021.Shareable
