# Supplementary material for: Reference equations for evaluation of spirometry function tests in South Asia, and among South Asians living in other countries
Source: Eur Respir J. 2022 Dec 1;60(6):2102962. doi: 10.1183/13993003.02962-2021 (PMC9712850; doi:10.1183/13993003.02962-2021)
Supplement: Supplementary file 1 [file ERJ-02962-2021.Supplement.pdf]

Supplementary tables

Table S1. Number of subjects categorised by exclusion criteria.

**Exclusion Criteria**	**Female**	**Male**	**Total**
**No. of subjects before exclusion**	27,285	16,187	43,472
***Current or ex-smokers**	375	6,420	6,795
***Lung disease: asthma, emphysema, chronic obstructive pulmonary disease (COPD), lung fibrosis, lung cancer or tuberculosis (TB)**	996	298	1,294
***Respiratory symptoms in the last 6 months: breathlessness during normal activity, persistent cough (at least 2 weeks), sputum during coughing, blood in the sputum, wheezing in the chest or early morning cough with chest tightness**	7,875	2,598	10,473
***Cardiovascular diseases: Angina pectoris, Myocardial Infarction, Stroke**	1,407	385	1,792
**Unavailable spirometry data or spirometry grading= D, E, F**	11,155	4,510	15,665
**No. of subjects after exclusion**	5,477	1,976	7,453

*Based on self-reported questionnaire

Table S2. List of South Asian spirometry equation studies published since 2005

	Location	Age (yrs)	Sample size	Remarks
**Bangladesh**				
Khuda et al. (2013)[1]	Bangladesh	18-40	995 healthy adults (628 males, 367 females)	Not included (No full-text)
**India**				
Saleem et al. (2012)[2]	Kashmir	18-65	3080 healthy adults (1974 males; 1106 females)	Included
Chhabra et al. (2014)[3]	Delhi	18-71	685 healthy adults (489 males; 196 females)	Included
Dasgupta et al. (2015)[4]	Kolkata	15-69	619 healthy adults (491 males, 128 females)	Included
Desai et al. (2016)[5]	Mumbai	18-75	310 healthy adults (185 males, 125 females)	Included
Biswas et al. (2018)[6]	Arunachal Pradesh	18-60	711 heathy adults (544 male, 167 females)	Included
Agarwal at al. (2020)[7]	Western India	>18 years	1258 healthy adults	Included
**Pakistan**				
Memon et al. (2007)[8]	Karachi	15-65	504 healthy adults (321 males; 183 females)	Included
**Sri Lanka**				
Sooriyakanthan et al. (2019)[9]	Northern Sri Lanka (Tamils)	14-85	775 males; 687 females	Included

[1]Khuda KM, Sultana R, Bari MR, Islam R, Erfan MA. Spirometric standard of healthy Bangladeshi adults aged 18-40 years. Mymensingh Med J. 2013 Jan;22(1):101-9.
[2]Saleem S, Shah S, Gailson L, et al. Normative Spirometric Values in Adult Kashmiri Population. Indian J Chest Dis Allied Sci 2012; 54:227–33.
[3]Chhabra SK, Kumar R, Gupta U, Rahman M, Dash DJ. Prediction equations for spirometry in adults from northern India. Indian J Chest Dis Allied Sci. 2014 Oct-Dec;56(4):221-9. Erratum in: Indian J Chest Dis Allied Sci. 2015 Jul-Sep;57(3):204.
[4]Dasgupta A, Ghoshal AG, Mukhopadhyay A, et al. Reference equation for spirometry interpretation for Eastern India. Lung India 2015.
[5]Desai U, Joshi JM, Chhabra SK, Rahman M-U. Prediction equations for spirometry in adults in western India. Indian J Tuberc 2016; 63: 176–82.
[6]Biswas Mrinmoy. Spirometry Prediction Equations for North-eastern Indian Population. Indian J Physiol Pharmacol 2018; : 431–8.
[7]Aggarwal A, Agarwal R, Dhooria S, et al. Joint Indian Chest Society-National College of Chest Physicians (India) guidelines for spirometry. Lung India 2019.
[8]Memon MA, Sandila MP, Ahmed ST. Spirometric reference values in healthy, non-smoking, urban Pakistani population. J Pak Med Assoc 2007; 57: 193–5.
[9]Sooriyakanthan M, Wimalasekera S, Kanagasabai S. Establishment of Reference Norms for Lung Function Parameters of Healthy Sri Lankan Tamils. Pulm. Med 2019

Table S3. List of South Asia reference equations used in this study

Publication	Sex	LFP	Predictive Equations	SEE
Saleem et al. (2012)	Female			
	15- 30 yrs	FEV_1	-0.468-0.015Age+0.023Heigh	0.442
	31-50 yrs		0.063-0.004Age+0.017Height	0.416
	>50 yrs		-1.356-0.002Age+0.024Height	0.410
	15- 30 yrs	FVC	0.244-0.022Age+0.022Height	0.454
	31-50 yrs		0.508-0.004Age+0.016Height	0.446
	≥50 yrs		-0.772-0.002Age+0.022Height	0.442
	15- 30 yrs	FEV_1/FVC(%)	67.8+0.137Age+0.105Height	3.139
	31-50 yrs		75.836-0.012Age+0.077Height	3.095
	≥50 yrs		54.976-0.021Age+0.205Height	3.166
	Male			
	15- 30 yrs	FEV_1	-1.136-0.014Age+0.033Height	0.627
	31-50 yrs		0.242-0.005Age+0.023Height	0.634
	≥50 yrs		-1.483-0.030Age+0.037Height	0.563
	15- 30 yrs	FVC	-0.416-0.021Age+0.032Height	0.685
	31-50 yrs		0.411-0.005Age+0.025Height	0.671
	≥50 yrs		-1.747-0.031Age+0.04Height	0.589
	15- 30 yrs	FEV_1/FVC(%)	72.742+0.106Age+0.089Height	2.891
	31-50 yrs		85.516-0.004Age+0.026Height	2.722
	≥50 yrs		84.987-0.085Age+0.047Height	3.537
Chhabra et al. (2014)	Female	FEV_1 (L)	-2.267 – 0.019(Age) + 0.033(Height)	0.286

		FVC (L)	20.07 – 0.010(Age) – 0.261(Height) + 0.000971($Height^2$)	0.315
		FEV_1/FVC (%)	97.182 – 0.440(Age)	4.97
	Male	FEV_1 (L)	-3.682 – 0.024(Age) + 0.046(Height)	0.402
		FVC (L)	-5.048 – 0.014(Age) + 0.054(Height) + 0.006(Weight)	0.479
		FEV_1/FVC(%)	102.56 – 0.679(Age) + 0.00477(Age^2) – 0.080(Weight)	5.79
Dasgupta et al. (2015)	Female	FEV_1 (L)	0.0381 – 0.0197(Age) + 0.0196(Height)	0.370
		FVC (L)	0.0972 – 0.0186(Age) + 0.0216(Height)	0.465
		FEV_1/FVC	0.9205 – 0.00214(Age) + 0.00001(Height)	0.076
	Male	FEV_1 (L)	-1.7649 – 0.0218(Age) + 0.0337(Height)	0.434
		FVC (L)	-2.5370 – 0.0211(Age) + 0.0418(Height)	0.518
		FEV_1/FVC	1.08994 – 0.00133(Age) – 0.0012(Height)	0.092
Desai et al. (2016)	Female	FEV_1 (L)	Exp[-1.552 + 0.0043(Age) – 0.000144(Age^2) + 0.015(Height)]	0.115
		FVC (L)	Exp[-1.616 + 0.014(Age) – 0.000219(Age^2) + 0.015(Height)]	0.097
		FEV_1/FVC(%)	104.35 – 0.085(Age) + 0.0065(Age^2)	6.34
	Male	FEV_1 (L)	-3.275 – 0.020(Age) + 0.043(Height)	0.346
		FVC (L)	Exp[-1.048 -0.0045(Age) + 0.015(Height)]	0.111
		FEV_1/FVC(%)	89.09 – 0.179(Age)	4.73
Biswas et al. (2019)	Female	FEV_1 (L)	0.770 – 0.021(Age) + 0.011(Height)	0.356
		FVC (L)	0.687 – 0.021(Age) + 0.014(Height)	0.359
		FEV_1/FVC	N/A	
	Male	FEV_1 (L)	0.269 – 0.025(Age) + 0.020 (Height)	0.460
		FVC (L)	0.003 – 0.024(Age) + 0.024 (Height)	0.448
		FEV_1/FVC	N/A	

Agarwal et al. (2020)	Female	FEV_1 (L)	-0.8376314 – 0.0095561(Age) + 0.0210584(Height)	0.30546
		FVC (L)	-1.217951 – 0.0061151(Age) + 0.0253754(Height)	0.36038
		FEV_1/FVC(%)	91.41677 – 0.2019615(Age) – 0.003514(Height)	5.7629
	Male	FEV_1 (L)	-0.6050618 – 0.0172575(Age) + 0.0249218(Height)	0.40664
		FVC (L)	-1.00747 – 0.0129677(Age) + 0.0299966(Height)	0.48185
		FEV_1/FVC(%)	88.87519 – 0.2001275(Age) + 0.0084023(Height)	5.056
Memon et al. (2007)	Female	FEV_1 (L)	-1.866 – 0.019(Age) + 0.032(Height)	0.480
		FVC (L)	-3.072 – 0.020(Age) + 0.042(Height)	0.611
		FEV_1/FVC	N/A	
	Male	FEV_1 (L)	-1.440 – 0.020(Age) + 0.030(Height)	0.427
		FVC (L)	-0.848 – 0.020(Age) + 0.032(Height)	0.508
		FEV_1/FVC	N/A	
Sooriyakanthan et al. (2019)	Female	FEV_1 (L)	− 1.186 - 0.018(Age) + 0.026(Height)	0.306
		FVC (L)	− 1.985 - 0.018(Age) + 0.033(Height)	0.364
		FEV_1/FVC	N/A	
	Male	FEV_1 (L)	− 2.192 - 0.021(Age) + 0.036(Height)	0.398
		FVC (L)	− 3.311 - 0.022(Age) + 0.046(Height)	0.469
		FEV_1/FVC	N/A	

Table S4. Summary of post hoc statistical test (tukey) for comparing differences in mean for FEV_1, FVC and FEV_1/FVC between countries (Bangladesh, India, Pakistan and Sri Lanka) *

	FEV_1 (L)		FVC (L)		FEV_1/FVC	
**Female**	**Mean diff.**	**p-value**	**Mean diff.**	**p-value**	**Mean diff.**	**p-value**
North India – Bangladesh	-0.11	<0.0001	-0.11	<0.0001	-0.01	<0.0001
South India – Bangladesh	-0.44	<0.0001	-0.61	<0.0001	0.06	<0.0001
Pakistan – Bangladesh	-0.12	<0.0001	-0.11	0.013	-0.01	0.058
Sri Lanka – Bangladesh	- 0.11	<0.0001	-0.18	<0.0001	0.02	<0.0001
South India – North India	-0.33	<0.0001	-0.50	<0.0001	0.06	<0.0001
Pakistan – North India	-0.01	0.999	0.00	1.000	-0.00	1.000
Sri Lanka – North India	-0.00	1.000	-0.07	0.001	0.03	<0.0001
Pakistan – South India	0.32	<0.0001	0.50	<0.0001	-0.06	<0.0001
Sri Lanka – South India	0.32	<0.0001	0.43	<0.0001	-0.03	<0.0001
Sri Lanka – Pakistan	0.01	0.999	-0.07	0.258	0.03	<0.0001
**Male**						
North India – Bangladesh	-0.13	0.001	-0.13	0.003	-0.01	0.325
South India – Bangladesh	-0.53	<0.0001	-0.70	<0.0001	0.02	0.044
Pakistan – Bangladesh	-0.04	0.982	0.03	0.998	-0.01	0.308
Sri Lanka – Bangladesh	-0.09	0.074	-0.16	0.001	0.01	0.010
South India – North India	-0.41	<0.0001	-0.57	<0.0001	0.02	0.001
Pakistan – North India	0.09	0.729	0.16	0.295	-0.01	0.823
Sri Lanka – North India	0.03	0.895	-0.03	0.959	0.02	<0.0001
Pakistan – South India	0.50	<0.0001	0.73	<0.0001	-0.03	0.006
Sri Lanka – South India	0.44	<0.0001	0.54	<0.0001	-0.00	0.991
Sri Lanka – Pakistan	-0.05	0.943	-0.19	0.169	0.03	0.004

**represents the differences in mean between 2 countries [eg. North India – Bangladesh= Mean of FEV_1 (North India) – Mean of FEV_1 (Bangladesh)]*

Table S5. Baseline characteristics of external study participants (HELIOS) by sex values are expressed in mean (SD), unless indicated otherwise (n=339).

	Female	Male
**n**	**214**	**125**
**Age (yrs)**	50.8 (11.1)	51.3 (10.5)
**Age group (yrs)**	%	%
30-39	18.7	12.8
40-39	24.3	32.0
50-59	36.0	34.3
≥ 60	21.0	20.8
**Height (cm)**	157.1 (5.9)	170.4 (5.9)
**Weight (kg)**	67.7 (14.1)	77.9 (11.9)
**FEV_1 (L)**	1.94 (0.44)	2.75 (0.55)
**FVC (L)**	2.36 (0.53)	3.38 (0.61)
**FEV_1/FVC (%)**	82.1 (5.4)	81.5 (5.9)

TableS6. Mean z-scores and % of subjects with z-scores below -1.645 in male (n=1976) and female (n=5477) using GLI2012 and NHANESIII reference values.

	**GLI 2012**								
**Ethnic**	**FEV$_1$ (L)**			**FVC (L)**			**FEV$_1$/FVC**		
**Others/Mixed**	*Predicted (SD)*	*z-score(SD)*	*z < -1.645 (%)*	*Predicted (SD)*	*z-score(SD*	*z < -1.645 (%)*	*Predicted (SD)*	*z-score(SD)*	*z < -1.645 (%)*
Male	3.16 (0.46)***	-1.43 (1.05)	39.9	3.85 (0.51)***	-1.46 (1.16)	41.7	0.82 (0.02)***	-0.20 (1.01)	8.1
Female	2.32 (0.34)***	-1.65 (1.04)	48.8	2.77 (0.36)***	-1.69 (1.18)	49.0	0.84 (0.03)***	-0.15 (1.04)	5.3
**African-American**									
Male	2.89 (0.42)***	-0.80 (1.06)	19.7	3.53 (0.46)***	-0.75 (1.10)	19.2	0.82 (0.02)***	-0.16 (0.96)	6.8
Female	2.14 (0.31)***	-1.08 (1.03)	27.4	2.58 (0.33)***	-1.06 (1.10)	26.5	0.83 (0.03)***	-0.07 (0.99)	4.2
**Caucasian**									
Male	3.39 (0.49)***	-1.86 (0.98)	58.2	4.18 (0.55)***	-1.89 (1.02)	58.6	0.81 (0.02)***	-0.05 (0.94)	5.3
Female	2.49 (0.36)***	-2.07 (0.98)	66.8	3.01 (0.39)***	-2.11 (1.05)	66.5	0.83 (0.03)*	0.00 (0.97)	3.2
**South-East Asian**									
Male	3.11 (0.45)***	-1.30 (1.05)	34.8	3.71 (0.49)***	-1.12 (1.10)	29.5	0.84 (0.03)***	-0.46 (1.02)	11.4
Female	2.20 (0.32)***	-1.27 (1.03)	33.9	2.59 (0.33)***	-1.12 (1.14)	28.9	0.85 (0.02)***	-0.42 (1.06)	9.4
	**NHANESIII**								
Ethnic	**FEV$_1$ (L)**			**FVC (L)**			**FEV$_1$/FVC**		
**Caucasian**	*Predicted (SD)*	*Mean (SD)*	*z < -1.645 (%)*	*Predicted (SD)*	*Mean (SD)*	*z < -1.645 (%)*	*Predicted (SD)*	*Mean (SD)*	*z < -1.645 (%)*
Male	3.38 (0.53)***	-2.05 (1.15)	63.3	4.26 (0.57)***	-2.28 (1.128)	72.2	0.79 (0.03)***	0.31 (1.08)	4.5
Female	2.50 (0.38)***	-2.23 (1.07)	70.8	3.06 (0.38)***	-2.36 (1.084)	75.4	0.81 (0.03)***	0.17 (1.07)	3.8
African-American									
Male	2.85 (0.45)***	-0.70 (1.06)	17.6	3.51 (0.47)***	-0.71 (1.12)	18.5	0.81 (0.02)*	-0.07 (0.99)	6.3
Female	2.06 (0.37)***	-0.76 (1.04)	18.3	2.49 (0.38)***	-0.78 (1.05)	18.5	0.83 (0.03)	-0.03 (0.98)	4.1

*Paired sample t-test (observed-predicted) p-value: ***<0.0001; **<0.001; *<0.01*

Table S7. Mean of predicted values, z-scores and % of subjects with z-scores below -1.645, in males (n=1976) and females (n=5477) using published South Asian reference values.

	Female			Male		
**FEV_1 (L)**	Predicted (sd)	z-score (sd)	z <-1.645 (%)	Predicted (SD)	z-score (sd)	z <-1.645 (%)
Observed	*1.81 (0.43)*	-	-	*2.55 (0.59)*	-	-
CHH	1.89 (0.34)***	-0.28 (1.15)	11.3	2.78 (0.48)***	-0.57 (1.15)	16.3
DAS	2.13 (0.30)***	-0.87 (0.87)	17.5	2.78 (0.39)***	-0.53 (1.04)	13.5
DES	1.85 (0.29)***	-0.37 (2.82)	30.1	2.87 (0.42)***	-0.94 (1.31)	26.3
BIS	1.50 (0.29)***	0.86 (0.93)	0.7	2.42 (0.37)***	0.28 (1.00)	4.2
AGA	1.93 (0.19)***	-0.39 (1.10)	12.5	2.70 (0.30)***	-0.39 (1.13)	12.2
MEM	2.14 (0.33)***	-0.68 (0.68)	8.3	2.58 (0.36)*	-0.07 (1.06)	7.2
SOO	1.95 (0.30)***	-0.47 (1.05)	12.7	2.76 (0.40)***	-0.55 (1.13)	15.5
SAL	2.40 (0.23)***	-1.41 (0.01)	36.1	3.49 (0.58)***	-1.52 (0.02)	43.5
**FVC (L)**						
Observed	*2.20 (0.51)*	-	-	*3.16 (0.70)*	-	-
CHH	2.40 (0.26)***	-0.65 (1.30)	20.4	3.57 (0.48)***	-0.87 (1.18)	23.3
DAS	2.54 (0.29)***	-0.74 (0.84)	13.3	3.36 (0.43)***	-0.40 (1.03)	10.4
DES	2.27 (0.30)***	-0.72 (4.15)	38.5	3.37 (0.42)***	-1.93 (4.88)	51.5
BIS	1.87 (0.29)**	0.91 (1.11)	1.7	2.86 (0.38)***	0.67 (1.23)	3.4
AGA	2.35 (0.18)***	-0.43 (1.19)	14.5	3.33 (0.29)***	-0.35 (1.16)	12.8
MEM	2.40 (0.39)***	-0.33 (0.64)	2.7	3.50 (0.37)***	-0.67 (1.06)	17.3
SOO	2.21 (0.33)*	-0.04 (1.07)	6.8	3.24 (0.46)***	-0.17 (1.14)	9.2
SAL	2.70 (0.24)	-1.12 (0.01)	26.1	3.86 (0.71)***	-1.05 (0.02)	28.8
**FEV_1/FVC**						
Observed	*0.82 (0.06)*	-	-	*0.81 (0.06)*	-	-
CHH	0.78 (0.06)***	0.96 (1.60)	3.7	0.77 (0.03)***	0.59 (1.17)	4.0
DAS	0.83 (0.03)**	-0.04 (0.84)	2.8	0.83 (0.02)**	-0.30 (0.68)	4.0
DES	1.15 (0.07)***	-5.07 (1.44)	99.7	0.81 (0.02)***	-0.10 (1.33)	11.0
AGA	0.82 (0.03)***	0.09 (1.10)	4.7	0.81 (0.03)***	-0.13 (1.26)	10.0
SAL	0.86 (0.01)***	-1.25 (0.03)	38.2	0.89 (0.01)***	-2.85 (0.05)	71.9

*1. Paired sample t-test (observed-predicted) p-value: ***<0.0001; **<0.001; *<0.01*

2. CHH= Chhabra, DAS= Dasgupta, DES= Desai, BIS= Biswas, AGA= Aggarwal, MEM= Memon, SOO= Sooriyakanthan, SAL= Saleem

Table S8. Table of characteristics for participants in development dataset (n=5589) and internal validation dataset (n=1864).

	Female		Male	
	Development	Internal validation	Development	Internal validation
**No. of subjects**	4121	1356	1468	508
**Age (yrs)**	44.5 (12.8)	44.5 (12.9)	45.3 (13.0)	44.1 (13.6)
**Age group (yrs)**	%	%	%	%
18-30	13.9	13.9	13.6	15.8
31-39	25.7	24.9	21.0	26.8
41-49	25.6	27.1	30.0	24.6
50-59	20.3	19.0	20.7	18.3
≥60	14.6	15.2	14.7	14.6
**Height (cm)**	151.5 (5.9)	151.5 (6.1)	163.9 (7.0)	163.8 (7.1)
**Weight (kg)**	59.4 (12.9)	59.7 (13.4)	67.0 (14.2)	66.6 (14.4)
**Countries**	%	%	%	%
Bangladesh	76.2	73.3	23.8	26.7
North India	68.1	70.3	31.9	29.7
South India	68.7	69.8	31.3	30.2
Pakistan	75.6	70.4	24.4	29.6
Sri Lanka	77.7	76.2	22.3	23.8

Table S9. Regression output for FEV_1, FVC and FEV_1/FVC stratified by sex (n=5589, female: n=4121; male: n=1468)

**FEMALE**									
	**FEV_1 (L)**			**FVC (L)**			**FEV_1/FVC**		
**VARIABLES**	**M1**	**M2**	**M3**	**M1**	**M2**	**M3**	**M1**	**M2**	**M3**
**Age**	-0.0173***	-0.0172***	-0.0167***	-0.0200***	-0.0199***	-0.0185***	-0.000209***	-0.000229***	-0.000605***
	(0.000399)	(0.000399)	(0.000403)	(0.000485)	(0.000482)	(0.000486)	(7.36e-05)	(7.25e-05)	(7.27e-05)
**Height**	0.0238***	0.0251***	0.0242***	0.0305***	0.0343***	0.0336***	-0.000628***	-0.00149***	-0.00158***
	(0.000868)	(0.000964)	(0.000939)	(0.00105)	(0.00117)	(0.00113)	(0.000160)	(0.000175)	(0.000169)
**Weight**		-0.00139***	0.0005		-0.00390***	-0.00148***		0.000877***	0.000765***
Study Region		(0.000432)	(0.000442)		(0.000523)	(0.000533)		(7.86e-05)	(7.97e-05)
**2.North India**			-0.169***			-0.172***			-0.0123***
			(0.0127)			(0.0153)			(0.00229)
**3.South India**			-0.296***			-0.432***			0.0473***
			(0.0212)			(0.0256)			(0.00383)
**4.Pakistan**			-0.204***			-0.199***			-0.0152***
			(0.0266)			(0.0321)			(0.00480)
**5.Sri Lanka**			-0.0679***			-0.139***			0.0246***
			(0.0132)			(0.0159)			(0.00238)
**Constant**	-1.027***	-1.152***	-1.060***	-1.533***	-1.883***	-1.859***	0.929***	1.008***	1.039***
	(0.136)	(0.141)	(0.138)	(0.165)	(0.170)	(0.166)	(0.0250)	(0.0256)	(0.0248)
**Observations**	4,121	4,121	4,121	4,121	4,121	4,121	4,121	4,121	4,121
**R-squared**	0.429	0.430	0.471	0.422	0.430	0.473	0.005	0.034	0.118
**N**	4121	4121	4121	4121	4121	4121	4121	4121	4121
**RSD**	0.323	0.322	0.311	0.392	0.390	0.375	0.0595	0.0586	0.0560
**Adjusted R-squared**	0.428	0.430	0.470	0.422	0.430	0.472	0.00444	0.0334	0.117
**MALE**									
	**FEV_1 (L)**			**FVC (L)**			**FEV_1/FVC**		
**VARIABLES**	**M1**	**M2**	**M3**	**M1**	**M2**	**M3**	**M1**	**M2**	**M3**
**Age**	-0.0215***	-0.0215***	-0.0210***	-0.0224***	-0.0224***	-0.0211***	-0.00122***	-0.00122***	-0.00145***
	(0.000909)	(0.000907)	(0.000912)	(0.00107)	(0.00107)	(0.00107)	(0.000126)	(0.000125)	(0.000128)
**Height**	0.0350***	0.0379***	0.0365***	0.0458***	0.0516***	0.0500***	-0.000703***	-0.00132***	-0.00134***

	(0.00169)	(0.00193)	(0.00188)	(0.00199)	(0.00227)	(0.00220)	(0.000234)	(0.000266)	(0.000263)
**Weight**		-0.00295***	-0.00109		-0.00575***	-0.00347***		0.000614***	0.000599***
Study Region		(0.000943)	(0.000946)		(0.00111)	(0.00111)		(0.000130)	(0.000133)
**2.North India**			-0.199***			-0.212***			-0.00988**
			(0.0283)			(0.0332)			(0.00397)
**3.South India**			-0.404***			-0.558***			0.0214***
			(0.0450)			(0.0527)			(0.00631)
**4.Pakistan**			-0.162**			-0.129*			-0.0172*
			(0.0644)			(0.0753)			(0.00902)
**5.Sri Lanka**			-0.0604*			-0.144***			0.0198***
			(0.0331)			(0.0387)			(0.00464)
**Constant**	-2.225***	-2.507***	-2.303***	-3.349***	-3.902***	-3.704***	0.976***	1.035***	1.049***
	(0.284)	(0.297)	(0.291)	(0.336)	(0.349)	(0.340)	(0.0394)	(0.0410)	(0.0407)
**Observations**	1,468	1,468	1,468	1,468	1,468	1,468	1,468	1,468	1,468
**R-squared**	0.429	0.432	0.471	0.422	0.433	0.476	0.063	0.077	0.114
**N**	1468	1468	1468	1468	1468	1468	1468	1468	1468
**RSD**	0.449	0.448	0.432	0.531	0.526	0.506	0.0622	0.0618	0.0606
**Adjusted R-squared**	0.428	0.431	0.469	0.421	0.431	0.474	0.0616	0.0751	0.110

M1: adjusted for age, height
M2: adjusted for age, height, weight
M3: adjusted for age, height, weight and study region (factorial variable)
****p<0.001, **p<0.01, *p<0.05*

Table S10. Internal validation: mean z-score and % predicted for M1, M2 and M3, stratified by study regions and sex (n=1864).

	FEMALE					
	FEV_1		FVC		FEV_1/FVC	
**Bangladesh**	**Mean z-score**	**% predicted**	**Mean z-score**	**% predicted**	**Mean z-score**	**% predicted**
M1	0.25	104.7	0.27	105.2	-0.10	99.3
M2	0.23	104.3	0.23	104.4	-0.04	99.7
M3	-0.02	99.8	-0.04	99.5	0.04	100.2
**North India**						
M1	-0.14	97.6	-0.04	99.3	-0.29	97.9
M2	-0.14	97.7	-0.03	99.7	-0.32	97.7
M3	0.11	102.0	0.12	102.0	-0.03	99.8
**South India**						
M1	-0.62	88.3	-0.85	83.6	0.88	106.4
M2	-0.59	88.9	-0.79	84.9	0.80	105.7
M3	0.01	100.7	-0.04	99.5	0.14	100.9
**Pakistan**						
M1	-0.25	95.5	-0.10	97.9	-0.42	96.9
M2	-0.21	96.2	0.00	99.4	-0.57	96.0
M3	0.09	101.3	0.14	101.8	-0.19	98.7
**Sri Lanka**						
M1	0.14	102.6	0.06	101.3	0.15	101.1
M2	0.14	102.7	0.07	101.7	0.13	100.9
M3	0.05	100.9	0.10	101.9	-0.16	98.9
	**MALE**					
	FEV_1		FVC		FEV_1/FVC	
**Bangladesh**	**Mean z-score**	**% pred**	**Mean z-score**	**% pred**	**Mean z-score**	**% pred**
M1	0.32	106.1	0.38	107.0	-0.12	99.1
M2	0.29	105.6	0.33	106.2	-0.08	99.4
M3	0.04	100.9	0.06	101.4	-0.06	99.6
**North India**						
M1	-0.21	97.9	-0.15	97.4	-0.19	98.5
M2	-0.20	97.7	-0.13	97.7	-0.21	98.4
M3	-0.03	99.8	-0.02	99.5	-0.03	99.7
**South India**						
M1	-0.43	106.4	-0.57	92.6	0.31	90.8
M2	-0.42	105.7	-0.56	92.9	0.30	91.3
M3	0.20	100.9	0.19	105.7	0.02	105.5
**Pakistan**						
M1	-0.32	94.5	-0.15	97.5	-0.24	98.2
M2	-0.29	95.3	-0.09	98.8	-0.31	97.7
M3	-0.23	96.3	-0.17	97.5	0.00	100.0
**Sri Lanka**						
M1	0.22	104.6	0.07	101.9	0.37	102.9
M2	0.23	105.0	0.10	102.5	0.35	102.7
M3	0.09	102.1	0.06	101.6	0.08	100.6

*1. One sided t-test of mean z-score=0; ***p<0.0001, **p<0.001, *P<0.01*

2. M1: adjusted for age, height; M2: adjusted for age, height, weight; M3: adjusted for age, height, weight, study region

Table S11. Internal validation: percentage of healthy subjects categorised below lower limit of normal (LLN) for FEV_1, FVC, FEV_1/FVC, stratified by reference equations and sex (n=1864).

	Female			Male		
**% < LLN**	**FEV_1 (L)**	**FVC (L)**	**FEV_1 /FVC**	**FEV_1 (L)**	**FVC (L)**	**FEV_1 /FVC**
**M1**	5.4	4.2	5.2	5.5	4.9	5.3
**M2**	5.2	4.3	5.4	5.7	5.3	5.3
**M3**	4.1	3.2	5.4	5.5	5.1	5.1
**CHH**	11.5	18.7	3.6	15.4	21.7	4.1
**DAS**	17.7	12.3	2.8	12.4	10.6	4.1
**DES**	30.3	37.1	99.8	25.2	50.6	11.0
**BIS**	0.4	1.0	-^	4.5	3.9	-^
**AGA**	11.6	13.0	4.9	11.2	12.0	10.4
**MEM**	8.3	2.0	-^	6.9	15.4	-^
**SOO**	12.5	5.5	-^	14.2	9.4	-^
**SAL1**	36.7	26.2	37.8	44.9	28.3	28.2
**NHA(AA)**	18.9	18.2	4.2	15.9	16.7	6.9
**GLI(AA)**	27.9	25.9	4.1	17.3	18.5	6.9

M1, M2, M3 are predictive equations from current study; CHH= Chhabra; DAS= Dasgupta; DES= Desai; BIS= Biswas; AGA= Aggarwal; MEM= Memon; SOO= Sooriyakanthan; SAL= Saleem; NHA(AA)= NHANES (African-American); GLI(AA)= GLI2012 (African-American)
Note:
-^spirometry reference equations are not available for measuring FEV_1/FVC

Table S12. Internal validation: Disease classification in healthy subjects, stratified by reference equations and sex (n=1864).

Female					Male				
	**Normal**	**Obstructive**	**Restrictive**	**Mixed**		**Normal**	**Obstructive**	**Restrictive**	**Mixed**
**M1**	91.2%	4.6%	3.5%	0.6%	**M1**	90.4%	4.7%	4.3	0.6
**M2**	91.0%	4.7%	3.6%	0.7%	**M2**	90.6%	4.1%	4.1%	1.2%
**M3**	91.9%	4.9%	2.7%	0.4%	**M3**	90.8%	4.1%	4.1%	1.0%
**CHH**	76.5%	2.5%	19.8%	1.1%	**CHH**	75.6%	2.8%	20.3%	1.4%
**DAS**	85.9%	1.8%	11.3%	1.0%	**DAS**	86.6%	2.8%	9.3%	1.4%
**DES**	0.0%	62.9%	0.2%	36.9%	**DES**	44.5%	4.9%	44.5%	6.1%
**SAL**	47.0%	26.8%	15.3%	10.9%	**SAL**	19.9%	51.8%	8.7%	19.7%
**SAL1**	50.5%	28.7%	11.7%	9.0%	**SAL1**	0%	80.0%	0.0%	20.0%
**AGA**	83.5%	3.5%	11.6%	1.4%	**AGA**	79.9%	8.1%	9.6%	2.4%
**NHA(AA)**	79.4%	2.4%	16.4%	1.8%	**NHA(AA)**	78.2%	5.1%	15.0%	1.8%
**GLI(AA)**	71.8%	2.3%	24.0%	1.8%	**GLI(AA)**	76.8%	4.7%	16.3%	2.2%

M1, M2, M3 are predictive equations from current study; CHH= Chhabra; DAS= Dasgupta; DES= Desai; BIS= Biswas; AGA= Aggarwal; MEM= Memon; SOO= Sooriyakanthan; SAL=Saleem; NHA(AA)= NHANES (African-American); GLI(AA)= GLI2012 (African-American)

Table S13. External validation (HELIOS): Percentage of healthy subjects categorised as below LLN (n=339, female: n=214; male: n=125).

	% of subjects below Lower Limit of Normal (LLN)					
	FEV_1 (L)		FVC (L)		FEV_1/FVC	
	Female	Male	Female	Male	Female	Male
**M1**	1.9	4.8	1.9	4.8	1.9	2.4
**M2**	1.9	4.8	0.9	4.8	2.3	2.4
**M3 (BGD)**	4.7	6.4	5.1	10.4	2.3	2.4
**M3 (NI)**	0.9	4.0	1.9	4.0	1.9	2.4
**M3 (SI)**	0.5	3.2	0.5	1.6	13.1	4.0
**M3 (P)**	0.5	4.8	1.4	6.4	1.9	2.4
**M3 (SL)**	3.3	6.4	1.9	6.4	4.7	4.0
**NHA (C)**	60.7	50.4	80.8	76.0	1.4	2.4
**NHA (AA)**	9.8	12.8	14.0	14.4	1.9	2.4
**GLI (C)**	57.0	47.2	61.2	56.8	1.4	2.4
**GLI (AA)**	14.9	13.6	20.6	12.8	1.9	2.4
**GLI (SEA)**	20.1	18.4	22.9	22.4	2.3	4.8
**GLI (OTH)**	36.4	21.6	39.7	36.0	1.9	2.4

M3(BGD) = Bangladesh, M3(NI)= North India, M3(SI) = South India, M3(P)= Pakistan, M3(SL)=Sri Lanka; NHA(C)= NHANES (Caucasian); NHA(AA)= NHANES (African-American); GLI(C)= GLI2012 (Caucasian); GLI(AA)= GLI2012 (African-American); GLI(SEA)= GLI2012 (South East Asian); GLI(OTH)= GLI (Others)

Supplementary figure

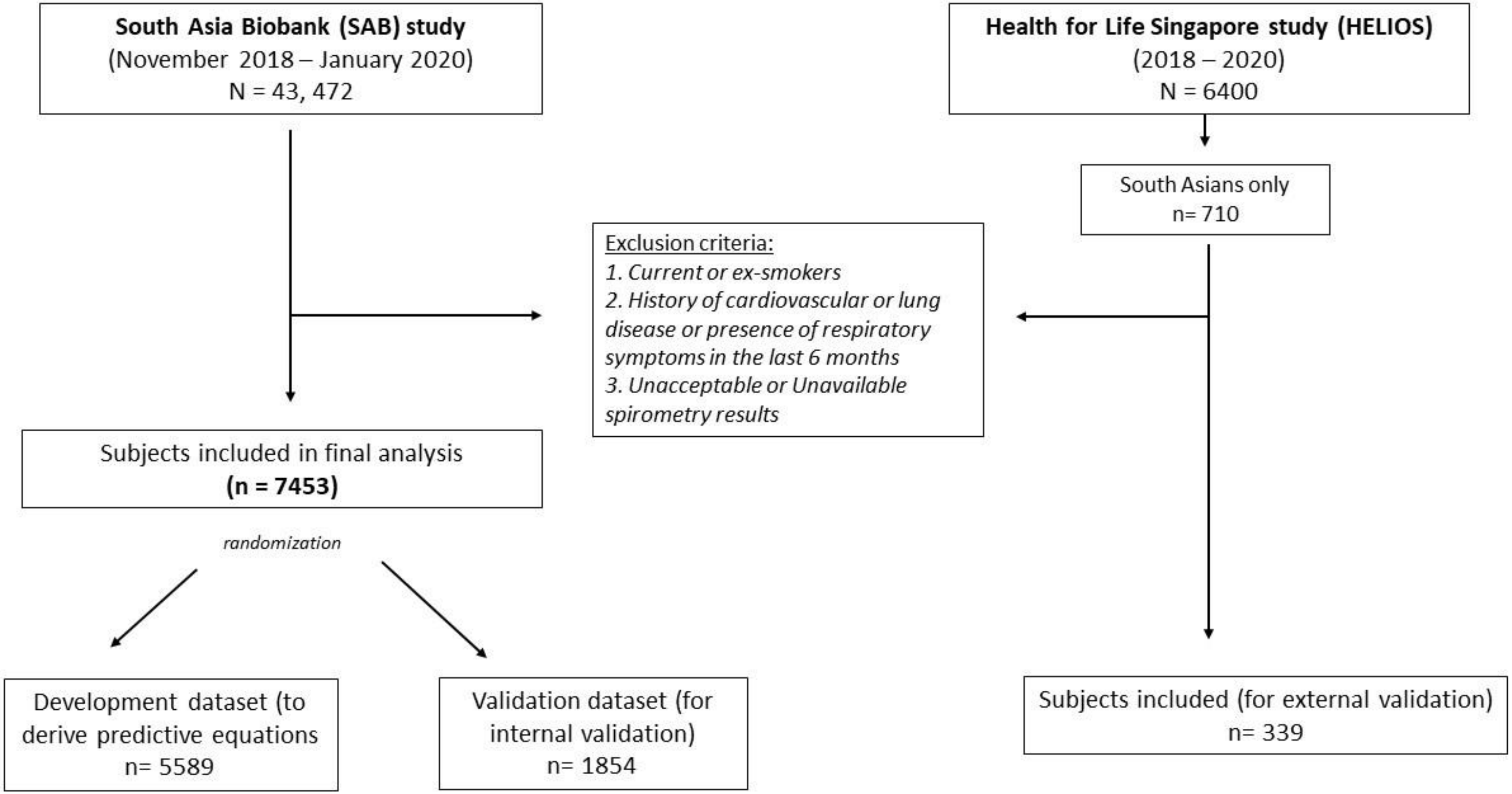

Figure S1. Flowchart of study design

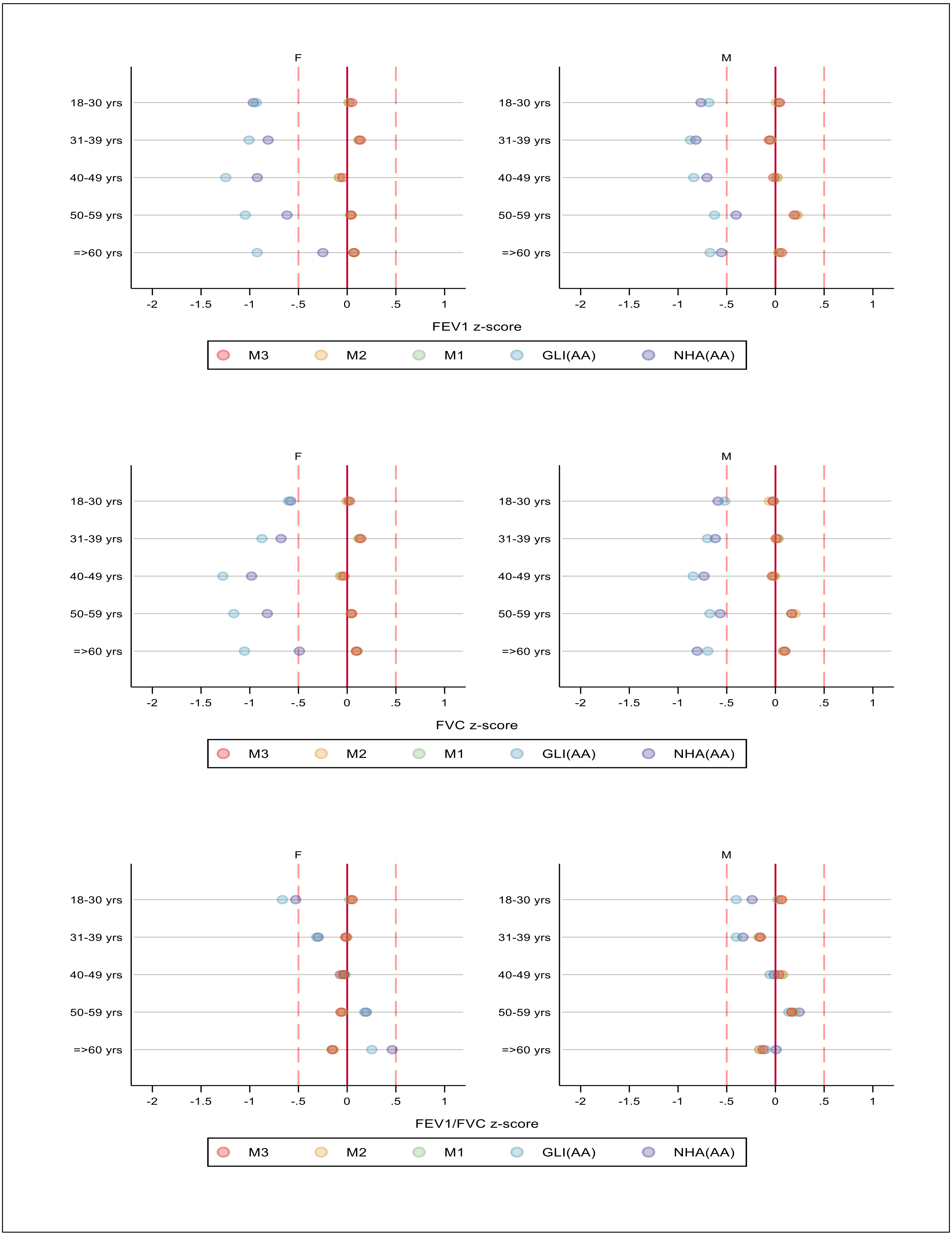

Figure S2. Mean z-score of FEV_1, FVC and FEV_1/FVC using GLI2012, NHANES and current predictive equations (M1, M2 and M3), stratified by age group and sex in internal validation (n=1864). *NHA(AA)= NHANES (African-American); GLI(AA)= GLI2012 (African-American).*

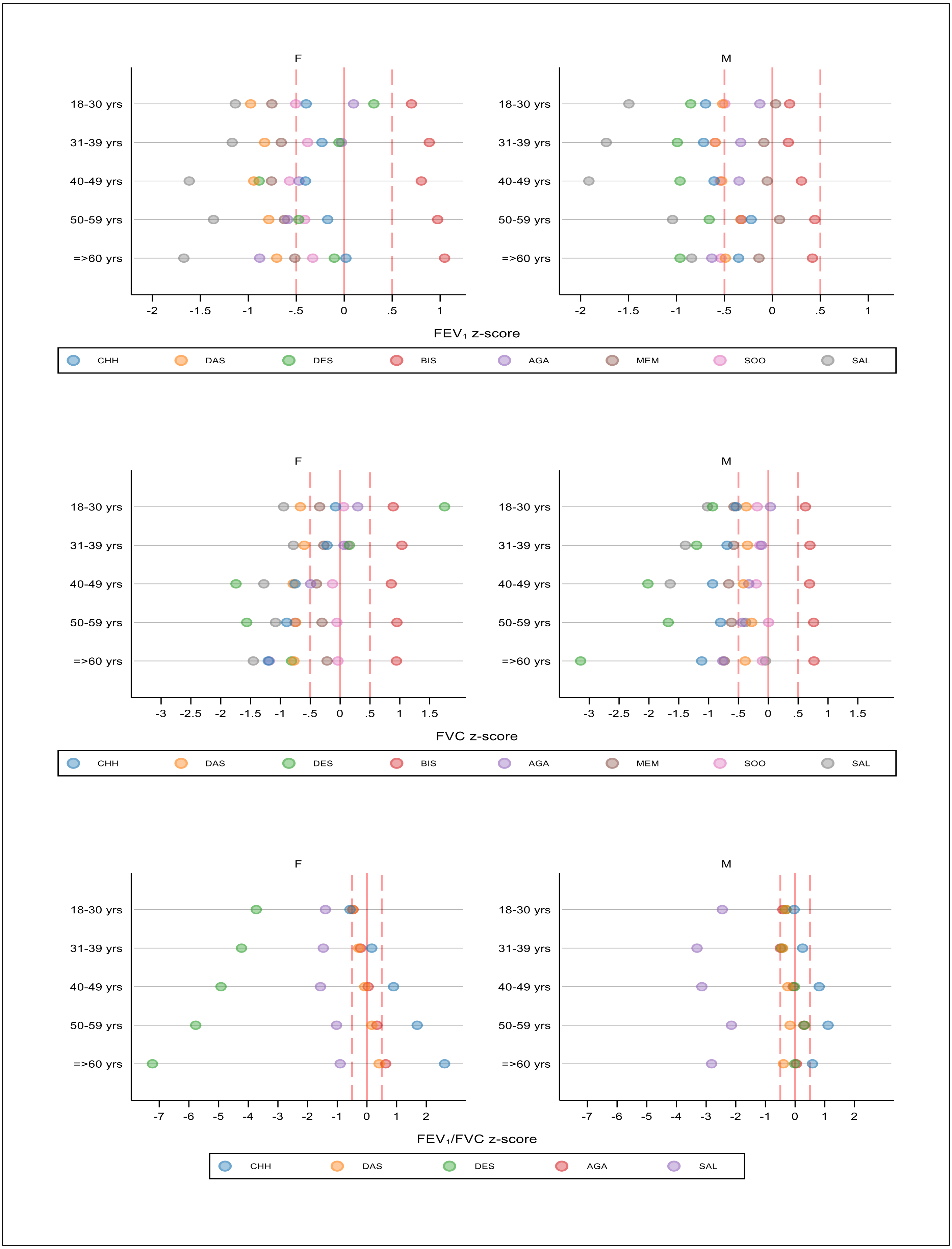

Figure S3. Mean z-score of FEV_1, FVC and FEV_1/FVC using South Asian published equations, stratified by age group and sex in internal validation (N=1864). (*CHH= Chhabra; DAS= Dasgupta; DES= Desai; BIS= Biswas; AGA= Aggarwal; MEM= Memon; SOO= Sooriyakanthan; SAL= Saleem)*
