# Supplementary material for: Reference equations for evaluation of spirometry function tests in South Asia, and among South Asians living in other countries
Source: Eur Respir J. 2022 Dec 1;60(6):2102962. doi: 10.1183/13993003.02962-2021 (PMC9712850; doi:10.1183/13993003.02962-2021)
Supplement: Supplementary file 2 [file ERJ-02962-2021.Shareable.pdf]

EUROPEAN RESPIRATORY JOURNAL
ORIGINAL RESEARCH ARTICLE
W.Y. LEONG ET AL.

SHAREABLE PDF

Reference equations for evaluation of spirometry function tests in South Asia, and among South Asians living in other countries

Wei Yee Leong[1], Ananya Gupta[2,3,4], Mehedi Hasan[5], Sara Mahmood[6], Samreen Siddiqui[3], Sajjad Ahmed[7], Ian Y. Goon[2,8], Marie Loh[1,2,9], Theresia H. Mina[1], Benjamin Lam[1,10], Yik Weng Yew[1,9], Joanne Ngeow[1,11], Jimmy Lee[1,12], Eng Sing Lee[13], Elio Riboli[2], Paul Elliott[2,14], Geak Poh Tan[15], Sanjay H. Chotirmall[1,15], Ananda R. Wickremasinghe[16], Jaspal S. Kooner[17], Khadija I. Khawaja[6], Prasad Katulanda[18], Malay K. Mridha[5], Sujeet Jha[3], Anjana Ranjit Mohan[19], Guha Pradeepa[19], Anuradhani Kasturiratne[16] and John C. Chambers[1,2]

[1]Lee Kong Chian School of Medicine, Nanyang Technological University, Singapore. [2]Department of Epidemiology and Biostatistics, School of Public Health, Imperial College London, London, UK. [3]Institute of Endocrinology, Diabetes and Metabolism, Max Super Speciality Hospital (Devki Devi Foundation), New Delhi, India. [4]Faculty of Medicine, Imperial College London, London, UK. [5]Centre for Non-communicable Disease and Nutrition (CNCDN), BRAC James P Grant of Public Health, BRAC University, Dhaka, Bangladesh. [6]Department of Endocrinology and Metabolism, Services Institute of Medical Sciences, Services Hospital, Lahore, Pakistan. [7]Punjab Institute of Cardiology, Punjab, Pakistan. [8]Tyree Foundation Institute of Health Engineering, The University of New South Wales, Sydney, Australia. [9]National Skin Centre, Singapore. [10]Department of Family and Community Medicine, Khoo Teck Puat Hospital, Singapore, Singapore. [11]Cancer Genetics Service, Division of Medical Oncology, National Cancer Centre Singapore, Singapore. [12]Institute of Mental Health, Singapore. [13]Clinical Research Unit, National Healthcare Group Polyclinics, Singapore. [14]MRC Centre for Environment and Health, Imperial College London, London, UK. [15]Department of Respiratory and Critical Care Medicine, Tan Tock Seng Hospital, Singapore. [16]Department of Public Health, Faculty of Medicine, University of Kelaniya, Ragama, Sri Lanka. [17]National Heart and Lung Institute, Imperial College London, London, UK. [18]Department of Clinical Medicine, Faculty of Medicine, University of Colombo, Colombo, Sri Lanka. [19]Madras Diabetes Research Foundation, Chennai, India.

Corresponding author: John C. Chambers (john.chambers@imperial.ac.uk)

Check for updates

Shareable abstract (@ERSpublications)
Newly derived spirometry reference equations greatly improve prediction of normal lung function in South Asians, thus filling the long-standing gap of appropriate reference values for this ethnic group https://bit.ly/3H7ZGt5

Cite this article as: Leong WY, Gupta A, Hasan M, *et al*. Reference equations for evaluation of spirometry function tests in South Asia, and among South Asians living in other countries. *Eur Respir J* 2022; 60: 2102962 [DOI: 10.1183/13993003.02962-2021].

This single-page version can be shared freely online.

Copyright ©The authors 2022.

This version is distributed under the terms of the Creative Commons Attribution Licence 4.0.

This article has an editorial commentary: https://doi.org/10.1183/13993003.01608-2022

Received: 16 Nov 2021
Accepted: 2 June 2022

Abstract

Background There are few data to support accurate interpretation of spirometry data in South Asia, a major global region with a high reported burden of chronic respiratory disease.

Method We measured lung function in 7453 healthy men and women aged ⩾18 years, from Bangladesh, North India, South India, Pakistan and Sri Lanka, as part of the South Asia Biobank study. First, we assessed the accuracy of existing equations for predicting normal forced vital capacity (FVC), forced expiratory volume in 1 s (FEV_1) and FEV_1/FVC ratio. Then, we used our data to derive (n=5589) and internally validate (n=1864) new prediction equations among South Asians, with further external validation among 339 healthy South Asians living in Singapore.

Results The Global Lung Initiative (GLI) and National Health and Nutrition Examination Survey consistently overestimated expiratory volumes (best fit GLI-African American, mean±SD z-score: FEV_1 −0.94±1.05, FVC −0.91±1.10; n=7453). Age, height and weight were strong predictors of lung function in our participants (p<0.001), and sex-specific reference equations using these three variables were highly accurate in both internal validation (z-scores: FEV_1 0.03±0.99, FVC 0.04±0.97, FEV_1/FVC −0.03±0.99) and external validation (z-scores: FEV_1 0.31±0.99, FVC 0.24±0.97, FEV_1/FVC 0.16±0.91). Further adjustment for study regions improves the model fit, with highest accuracy for estimation of region-specific lung function in South Asia.

Conclusion We present improved equations for predicting lung function in South Asians. These offer the opportunity to enhance diagnosis and management of acute and chronic lung diseases in this major global population.
